# Indexing Effects of Copy Number Variation on Genes Involved in Developmental Delay

**DOI:** 10.1038/srep28663

**Published:** 2016-07-01

**Authors:** Mohammed Uddin, Giovanna Pellecchia, Bhooma Thiruvahindrapuram, Lia D’Abate, Daniele Merico, Ada Chan, Mehdi Zarrei, Kristiina Tammimies, Susan Walker, Matthew J. Gazzellone, Thomas Nalpathamkalam, Ryan K. C. Yuen, Koenraad Devriendt, Géraldine Mathonnet, Emmanuelle Lemyre, Sonia Nizard, Mary Shago, Ann M. Joseph-George, Abdul Noor, Melissa T. Carter, Grace Yoon, Peter Kannu, Frédérique Tihy, Erik C. Thorland, Christian R. Marshall, Janet A. Buchanan, Marsha Speevak, Dimitri J. Stavropoulos, Stephen W. Scherer

**Affiliations:** 1 The Centre for Applied Genomics, The Hospital for Sick Children, Toronto, Ontario, Canada; 2 Program in Genetics and Genome Biology (GGB), The Hospital for Sick Children, Toronto, Ontario, Canada; 3 Department of Molecular Genetics, University of Toronto, Toronto, Ontario, Canada; 4 Center of Neurodevelopmental Disorders (KIND), Neuropsychiatric Unit, Department of Women’s and Children’s Health, Karolinska Institutet, Stockholm, Sweden; 5 Center for Human Genetics, University of Leuven, Leuven, Belgium; 6 CHU Sainte-Justine, University de Montreal, Montreal, Quebec, Canada; 7 Genome Diagnostics, Paediatric Laboratory Medicine, The Hospital for Sick Children, Toronto, Ontario, Canada; 8 Department of Pathology and Laboratory Medicine, Division of Diagnostic Medical Genetics, Mount Sinai Hospital, Toronto, Ontario, Canada; 9 Department of Genetics, The Children’s Hospital of Eastern Ontario, Ottawa, ON, Canada; 10 Division of Clinical and Metabolic Genetics, Department of Pediatrics, The Hospital for Sick Children, University of Toronto, Toronto, Ontario M5G 2L3, Canada; 11 Cytogenetics Laboratory, Department of Laboratory Medicine and Pathology, Mayo Clinic, Rochester, Minnesota, USA; 12 Department of Laboratory Medicine and Pathobiology, University of Toronto, Toronto, Ontario, Canada; 13 McLaughlin Centre, University of Toronto, Toronto, Ontario, Canada

## Abstract

A challenge in clinical genomics is to predict whether copy number variation (CNV) affecting a gene or multiple genes will manifest as disease. Increasing recognition of gene dosage effects in neurodevelopmental disorders prompted us to develop a computational approach based on critical-exon (highly expressed in brain, highly conserved) examination for potential etiologic effects. Using a large CNV dataset, our updated analyses revealed significant (P < 1.64 × 10^−15^) enrichment of critical-exons within rare CNVs in cases compared to controls. Separately, we used a weighted gene co-expression network analysis (WGCNA) to construct an unbiased protein module from prenatal and adult tissues and found it significantly enriched for critical exons in prenatal (P < 1.15 × 10^−50^, OR = 2.11) and adult (P < 6.03 × 10^−18^, OR = 1.55) tissues. WGCNA yielded 1,206 proteins for which we prioritized the corresponding genes as likely to have a role in neurodevelopmental disorders. We compared the gene lists obtained from critical-exon and WGCNA analysis and found 438 candidate genes associated with CNVs annotated as pathogenic, or as variants of uncertain significance (VOUS), from among 10,619 developmental delay cases. We identified genes containing CNVs previously considered to be VOUS to be new candidate genes for neurodevelopmental disorders (*GIT1*, *MVB12B* and *PPP1R9A*) demonstrating the utility of this strategy to index the clinical effects of CNVs.

The broad umbrella classification of “developmental disorders” encompasses various conditions characterized by disturbance or delay of developmental milestones that appear in infancy or childhood. The cluster includes diagnostic entities that are themselves collectives, such as autism spectrum disorder (ASD), intellectual disability, learning disability, and others. The term may be used rather loosely, but is a common reason for referral to laboratories that offer genomic microarray for diagnostic evaluation. Developmental disorders affect ~3% of the population, and reflect a significant genetic contribution^1^,^2^,^3^. In particular, large-scale genome-wide investigations^3^,^4^,^5^,^6^,^7^,^8^,^9^ have demonstrated the large impact of copy number variation (CNV) on severe pediatric conditions, including various developmental disorders. As a result, whole genome “chromosomal microarray” (CMA) has come to be used as a first tier diagnostic test to elucidate causal CNVs in individuals with developmental disorders and congenital anomalies^3^,^7^,^10^. In a pediatric genetics laboratory, the variants detected by CMA are interpreted with respect to probable clinical significance, based on variant type (deletion or duplication), inheritance (*de novo* or present in a parent), gene content, gene density and evidence from published literature on association with diseases. For large, rare recurrent deletions and duplications (e.g., 16p11.2, 22q11.2, 15q13.2), the interpretation is rather obvious due to overwhelming genetic and phenotypic evidence. In a large multi-centre clinical data set, 15–20% of cases with developmental delay were associated with diagnostic findings on clinical chromosomal microarray^3^.

Among these diagnostic cases, many rare CNVs are detected for which the potential functional significance is unknown, and are referred to as variants of uncertain (or unknown) significance (VOUS). Variants deemed to be of clinical significance (or pathogenic) are fewer, and not all genes impacted by these variants will influence the developmental disability phenotype. Few candidate genes ascertained from clinically significant CNVs have been established as pathogenic through genetic studies; some are supported by model organism experiments^6^,^11^,^12^,^13^,^14^,^15^. The more abundant VOUS remain largely uncharacterized, and we need to investigate their expression and regulatory networks to understand their contribution to human cognition, behavior and disease. By virtue of the evaluation criteria, these variants are usually shorter than those classified as significant; they impact fewer exons or genes, and are mostly unique observations among cases studied^3^,^7^. A typical gene-level case-control association analysis of rare variants reveals thousands of genes to be apparently significant (Coe *et al.*^6^ reported ~3800 genes *P* < 0.01)^6^ but these include many spurious signals that arise due to physical proximity to truly significant genes, given that large CNVs encompass multiple genes.

Genome-wide molecular data (transcriptomic, proteomic, etc.) facilitate inference of pathogenic mechanisms through information about expression and regulatory networks and pathways at the molecular and cellular level^16^,^17^,^18^,^19^. Investigations of existing biological networks are biased towards the number of simplified interactions^20^ or do not take tissue specificity into account^21^,^22^, yet these are important factors for the elucidation of phenotypically relevant genes^23^. The aim of this study was to assess the probable impact of variants in genes from within CNVs–both those established as clinically significant and VOUS–through knowledge of mutational burden, and RNA and protein expression in various human brain tissues at different developmental stages (prenatal and adult). Previously, we developed a robust method, coupling information about the burden of exonic mutations with exon-level expression, to quantify the critical nature of an exon in a given tissue at specified times in development (hence, spatio-temporal expression)^24^. We revealed an inverse correlation between exon expression level in brain and the burden of rare missense mutations found in population controls. Variants in these specific critical exons are significantly enriched among individuals with autism, relative to their unaffected siblings. Building upon this concept, we have now implemented an integrated genomics approach to analyze CMA data from DNA of individuals with developmental delay, to infer biologically relevant genes at the transcriptome and proteome levels. For this analysis, we added RNA-seq data from 388 postmortem brain tissues (prenatal to adult) and re-constructed the exon transcriptome contingency index for 226,845 exons from 19,631 genes. We identified ‘brain critical exons’ with i) a low burden (<75^th^ percentile for the genome) of rare (<5%) missense and loss-of-function (LOF) mutations as identified from the 1,000 Genomes Project^25^, and ii) high expression (>75^th^ percentile for the genome) in brain tissue. We utilized these data to quantify the enrichment of ‘critical exons’ in 16 brain regions^16^. Next, we used genome-wide indexing of critical exons to quantify genes impacted by pathogenic or VOUS in developmental delay cases, and rare CNVs in controls. We used a comprehensive high resolution proteome dataset from prenatal and adult tissues (16) to analyze and extract biologically relevant gene co-expression networks. New candidate genes from within the pathogenic variants and VOUS were inferred from the aggregate analysis of spatio-temporal mRNA and protein expression data.

## Results

### Pathogenic variants and variants of uncertain significance

**Figure 1:**
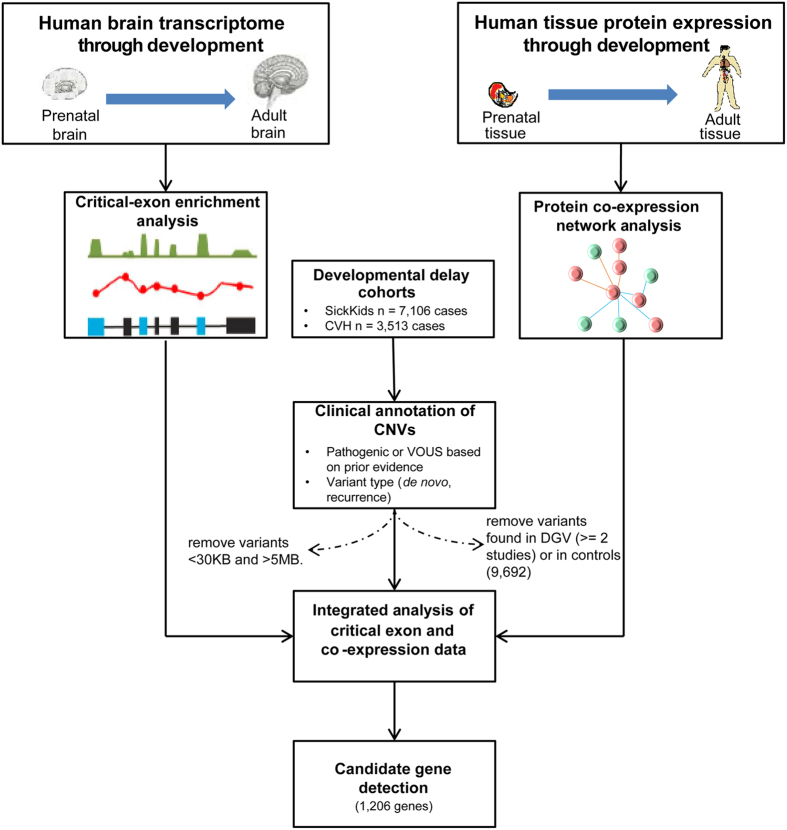
Schematic of the analysis framework to identify candidate genes from copy number variation in developmental delay, using genome-wide human (prenatal and adult) brain transcriptome (RNA sequencing) and proteome data (Fourier-transform mass spectrometry). The spatio-temporal transcriptome data was used to compute ‘brain critical exon’ analysis for all the genes in the genome. Quantified protein expression for each gene in the genome was used for weighted gene co-expression network analysis. To identify a candidate set of phenotypically relevant genes, integrated (transcriptome and proteome) analysis was conducted for genes impacted by rare CNVs in cases (pathogenic/VOUS) and controls. CVH, Credit Valley Hospital; VOUS, variant of unknown significance; DGV, Database of Genomic Variants.

**Figure 2:**
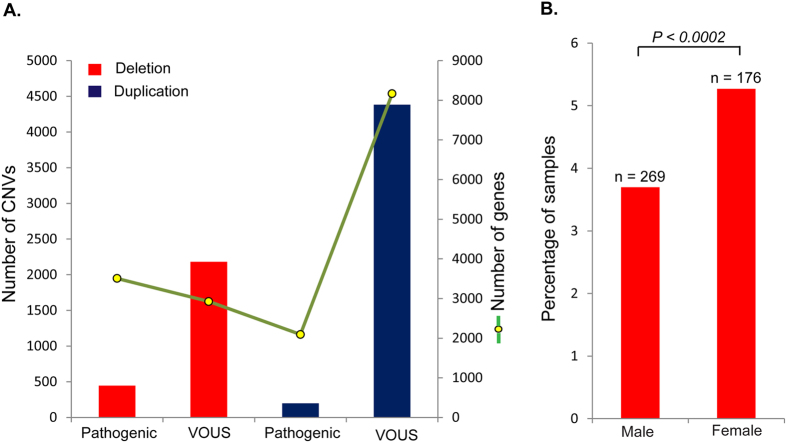
Ascertainment of pathogenic variants or variants of uncertain significance (VOUS) in 10,619 developmental delay cases. The rare CNVs of 30 kb to 5 Mb were classified as pathogenic variants or of VOUS. (**A**) Bars indicate the total number of CNVs in each classification. Of all samples assayed, 4.19% carried a pathogenic deletion, 1.81% a pathogenic duplication, 18.28% a VOUS deletion and 31.97% a VOUS duplication. The green line represents the number of unique genes impacted by the corresponding variants. (**B**) The percentage of male and female cases in the cohort impacted by pathogenic deletion variants. *P* value shown is for the one-sided Fisher’s exact test.

We analyzed 10,619 cases from two Ontario hospitals (The Hospital for Sick Children (SickKids) and Credit Valley Hospital (CVH)) referred for clinical laboratory testing due to developmental delay. We also used 9,692 controls (described in Methods) for whom no known psychiatric condition has been reported (Fig. 1 and Tables S1–S2). From this developmental delay cohort, 10.15% of the samples carried a pathogenic CNV, and 50.25% had at least one rare VOUS (Fig. 2A). In the SickKids subset (7,106 cases), which included inheritance information, there were 169 *de novo* variants (108 deletions and 61 duplications), of which 64% (108/169) were interpreted as pathogenic and 36% (61/169) as VOUS. To reduce platform specific sensitivity and specificity bias, we restricted our analysis to variants of 30 Kb to 5 Mb in length. The developmental delay cohort was 68.53% male and 31.46% female. There were pathogenic deletion variants in 5.26% of females and 3.69% of males, and this difference was highly significant (P < 0.0002) (Fig. 2B). This sex difference was not found for duplication CNVs (Fig. S1). The average number of genes with exonic variants differed significantly between pathogenic CNVs and VOUS. Genes per variant averaged 38 for pathogenic duplications, 27 for pathogenic deletions, 4.6 for VOUS duplications, and 3.2 for VOUS deletions (Fig. S2). The average number of genes in pathogenic deletions from females was higher than in those from males (Fig. S3). CNVs at loci for known genomic disorders^26^ were more frequent in our developmental delay cohort relative to controls, for example 16p11.2 (0.86%), 15q13.3 (0.43%), Prader-Willi syndrome (0.52%) and 22q11 deletion/duplication syndrome (1.06%) (Fig. S4).

### Critical Exon Analysis

**Figure 3:**
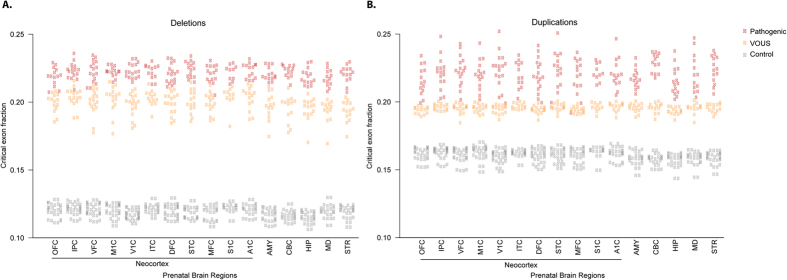
The fraction of critical exons (over all exons) computed from human prenatal brain regions for the genes impacted by pathogenic, VOUS and rare control deletion and duplication variants. (**A**,**B**) The critical exon fraction was computed using gene expression level quantified from RNA sequencing in 392 brain tissues (controls) from 31 postmortem donors in 2 developmental periods (prenatal and adult) for 16 brain regions (AMY, amygdaloid complex; CBC, cerebellar cortex; V1C, primary visual cortex; STC, posterior (caudal) superior temporal cortex; IPC, posterior inferior parietal cortex; A1C, primary auditory cortex; S1C, primary somatosensory cortex; M1C, primary motor cortex; STR, striatum; DFC, dorsolateral prefrontal cortex; MFC, medial prefrontal cortex; VFC, ventrolateral prefrontal cortex; OFC, orbital frontal cortex; MD, mediodorsal nucleus of thalamus; ITC, inferolateral temporal cortex; HIP, hippocampus). The critical exon fraction was computed using prenatal brain transcriptome for the genes impacted by pathogenic (red dots) or VOUS (orange dots) or rare control deletions (gray dots).

Genes within pathogenic or VOUS CNVs in this developmental delay cohort had a significantly higher fraction of critical exons (computed over all exons impacted by CNVs), compared with genes in the rare duplications or deletions seen in controls (Table S2). Gene sets from pathogenic CNVs and VOUS were very large, and overlapped those from control CNVs; we limited the analysis to genes impacted by pathogenic CNVs or VOUS from the case cohort, and not found in unaffected controls (Fig. 3). We observed a striking enrichment of critical exons (computed using prenatal brain transcriptome) within pathogenic deletions (corrected Fisher’s Exact Test (FET) with P < 1.64 × 10^−15^ for a brain region) and VOUS deletions (P < 1.31 × 10^−20^ to 6.22 × 10^−158^) (Fig. 3B). This result strongly suggests that pathogenic and VOUS variants harbor more critical exons than do the rare CNVs in controls. Although deletions showed the consistently highest sensitivity, we observed a similar increased critical exon fraction for pathogenic and VOUS duplications computed using prenatal and adult transcriptome (Fig. 3B and Fig. S5). We then used these exclusively pathogenic and VOUS gene sets to identify candidates for effects on developmental disorders. Of interest, the fraction of critical exons was highest in prenatal neocortical regions (specifically medial prefrontal cortex (MFC) tissues) for genes impacted by either pathogenic or VOUS deletions. This observation strongly supports previous independent reports of multiple neuropsychiatric patients with phenotypes associated with MFC^27^,^28^,^29^.

### Protein co-expression analysis

**Figure 4:**
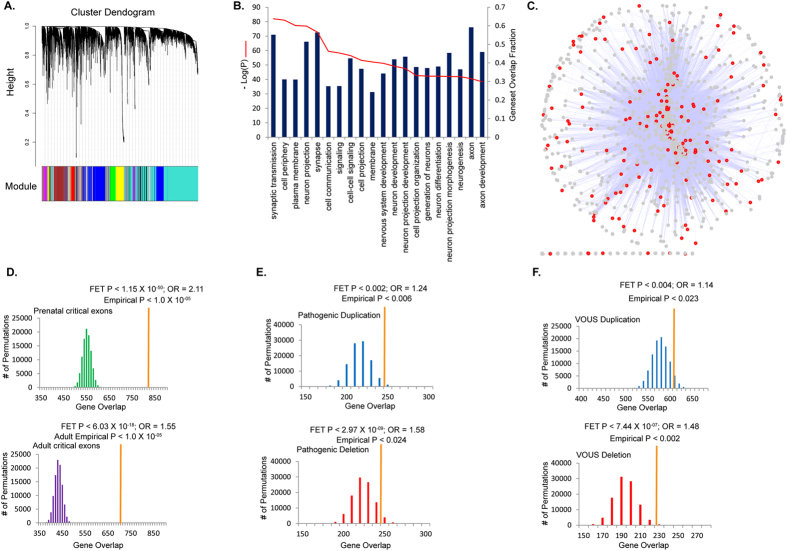
Genome-wide protein co-expression and enrichment of genes ascertained from pathogenic and VOUS CNVs using human prenatal and adult tissues. (**A**) The protein modules generated by weighted gene coexpression network analysis (WGCNA) using high resolution genome-wide Fourier-transform mass spectrometry data from 30 histologically normal human samples (prenatal and adult). Each colour (bars dispersed) represents a module. (**B**) For the blue module, the 20 most significant results from quantitative association with 18,826 gene sets. (**C**) Representation of the ‘blue’ module as a functional network, where each node is a gene; the edge between genes represents the weighted Pearson distance. Red nodes represent genes ascertained through CNVs in the developmental delay cohort. (**D**) The top (25^th^ percentile) critical exon genes in the genome ascertained using prenatal (green) and adult brain (purple) transcriptomes, and their corresponding quantified enrichment in the protein module, through Fisher’s exact test (FET) and 100,000 permutations. The orange bar represents the original observation of overlaps between blue module and a gene set. Similarly, (**E**,**F**) show the enrichment of genes impacted by pathogenic and VOUS duplications (blue) and deletions (red) within the protein module.

Expression of mRNA is not highly correlated with protein expression, even within the same cells under similar conditions, due to many biological processes that impact mRNA prior to protein translation. Integrated analysis of transcriptome and proteome will provide more potent evidence of gene expression within a tissue. To examine the biological relevance of pathogenic and VOUS ‘critical exons’ at the protein level, we applied co-expression analysis using Fourier transformed protein expression data. We used a draft of the human proteome map (HPM) reported by Kim *et al.*^19^, obtained from mass spectrometry of 24 different human tissues (each pooled from 3 post-mortem samples) including 17 adult and 7 prenatal^19^. To our knowledge, ours is the most comprehensive human developmental (prenatal and adult) protein co-expression analysis using this high resolution Fourier transformed dataset. Unlike the biased seeding approach used to construct modules and networks^30^,^31^,^32^, we implemented an unbiased construction of networks, based on spatio-temporal protein expression, by applying a weighted gene co-expression network analysis (WGCNA)^33^. We applied WGCNA to the 17,294 genes represented by the proteome^19^ to construct protein co-expression networks. We excluded genes not expressed in at least 90% of the tissues. The analysis revealed networks for 23 independent protein expression modules (Fig. 4A). To identify modules that are relevant to developmental delay, we conducted gene enrichment analysis using 18,826 gene ontology (GO) terms, and retained the 20 most significant GO terms. From this analysis, we identified the ‘blue’ module (Fig. 4A), which comprised 2,484 genes (Table S4), and was highly significant (*P* < *1.0* × *10*^*−39*^ to *1.0* × *10*^*−81*^) for pathways involved in synaptic transmission, neuron projection, cell signaling, nervous system development and axon guidance (Fig. 4B).

### Identification of candidate developmental delay genes from integrated analysis

To develop a list of candidate genes related to developmental delay, we combined the two approaches - critical exon and WGCNA core protein–analyzing genes from the protein-derived ‘blue’ module for critical exon enrichment. For each gene in the genome, we computed the critical exons for prenatal and for adult tissues. To control for the genes with a large number of exons, for each gene in the genome, we computed the fraction of critical exons (over all exons) for a gene in each brain region. For each developmental period (prenatal or adult), a gene was deemed to be significant if its critical exon fraction fell within the genome’s top 25^th^ percentile for at least 50% of the brain samples (Table S6). We found 48.5% (1,206/2,484) of ‘blue’ module proteins to be within the top 25^th^ percentile of genes enriched for critical exons, both for prenatal (FET, *P* < 1.15 × 10^−50^, OR = 2.11) and adult brain (FET, *P* < 6.03 × 10^−18^, OR = 1.55) (Table S5). This included genes (*SCN2A, NRXN1, NRXN2, NRXN3, SHANK2, NLGN3, STXBP1, NLGN4*) known to be associated with neuropsychiatric conditions^34^,^35^. Inference of these 1,206 candidate genes, as described, was independent of the knowledge of gene-disease association. The overrepresentation of critical exon genes within the ‘blue’ module was tested by random permutation 100,000 times to obtain an empirical significance (for both periods, *P* < 1.0 × 10^−05^) (Fig. 4C,D) using the appropriate background (please see methods).

We then tested from within the ‘blue’ module for overrepresentation in tiers of genes that are associated with certain neurodevelopmental disorders. There was significant enrichment of the ‘blue’ module with 840 fragile X mental retardation protein (FMRP) target genes^36^ (P < 1.36 × 10^−128^, OR = 6.25; empirical P < 1.0 × 10^−05^), 246 genes impacted with *de novo* LOF mutations in ASD cases^35^,^37^,^38^,^39^,^40^ (P < 5.9 × 10^−04^, OR = 1.79; empirical P < 2.1 × 10^−04^) and 141 genes that had *de novo* exonic deleterious mutations in intellectual disability samples^41^,^42^ (P < 8.0 × 10^−03^; OR = 1.80; empirical P < 2.3 × 10^−03^) from exome and whole genome sequencing (Fig. S6A–C). This result suggested that proteins in this ‘blue’ module are under purifying selection in brain, and that perturbing them may lead to neurodevelopmental phenotypic manifestations.

Next, we looked for ‘blue’ module genes among those ascertained from CNVs in the developmental delay cohort. ‘Blue’ module genes were significantly overrepresented among genes within pathogenic deletions (P < 2.97 × 10^−09^; OR = 1.58; empirical P < 0.024) or duplications (P < 0.002; OR = 1.24; empirical P < 0.006) (1-sided FET and permutation test). They were similarly overrepresented among genes within VOUS deletions (P < 7.44 × 10^–07^; OR = 1.48; empirical P < 0.002) and duplications (P < 0.004; OR = 1.14; empirical P < 0.023) (Fig. 4E,F). In contrast, the genes within deletions and duplications in controls were not overrepresented within the ‘blue’ module. We found 438 candidate genes (of 1,206 genes) that were included in the ‘blue’ module and also highly enriched for critical exons (top 25^th^ percentile), that had been ascertained by at least one pathogenic variant or VOUS in the developmental delay cohort (Table S5). Of these, 65 (14.84%) were seen in at least 2 VOUS deletions in cases but not controls. Another 37 of these 438 genes were impacted by at least one *de novo* VOUS in our dataset (Table S5). Genes from large recurrent CNVs known to be associated with neuropsychiatric syndromes (e.g., 16p11.2, 22q11, and 3q29)^43^ were represented in the ‘blue’ protein module and were highly enriched for ‘critical exons’ in prenatal or adult brain (Table S6).

### New candidate gene: *GIT1*

**Figure 5:**
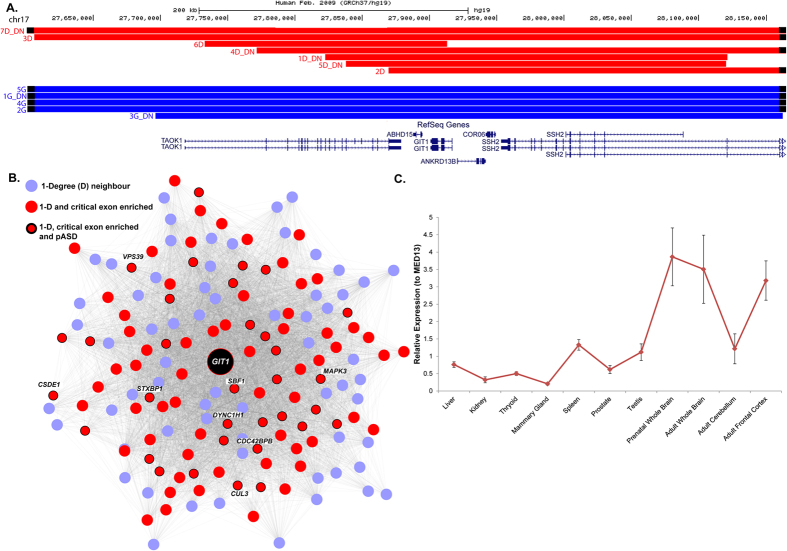
Deletions within the *GIT1* gene identified in developmental disorder cases or controls. (**A**) The breakpoints of 12 VOUS deletions (red) and duplications (blue) impacting *GIT1* and nearby genes. The dataset includes 5 *de novo* deletions/duplications (denoted as DN) reported from developmental cases; no rare CNV was found in controls. (**B**) The analysis of the human protein co-expression network revealed that *GIT1* is within the blue module and is highly connected (1-degree neighbors) with genes enriched for ‘critical exons’ (red nodes) and putative ASD genes reported to have *de novo* mutations (red node with black outline). (**C**) Expression of *GIT1* (primer targeting critical exons) from quantitative real-time PCR (qRT-PCR) relative to housekeeping gene, *MED13* (replicated with another housekeeping gene *ACTB*) in 11 different tissues.

Through unbiased critical exon analysis coupled with WGCNA we identified 1,206 candidate genes whose disruption is likely to contribute to neurodevelopmental delay (Table S5). For example, in our cohort, VOUS deletions and duplications within chromosome region17q11.2 affect the gene for G protein-coupled receptor kinase interacting ArfGAP1 (*GIT1*). Within the VOUS in this region, *GIT1* was the only gene enriched with critical exons in prenatal brain, and was highly clustered with genes (highly connected first degree neighbors) from within the ‘blue’ protein module network that were reported to have *de novo* mutations in ASD. The *GIT1* protein is involved in cell migration^44^, localizes in pre- and post-synaptic terminals, and regulates synapse formation^45^. Recent studies of knock-out Git1−/− mice showed a decreased brain size, with impaired motor coordination and deficits in learning and memory^46^. From published data^6^ and on additional clinical cohorts (Mayo clinic, DatabasE of genomiC varIation and Phenotype in Humans using Ensembl Resources (DECIPHER) and Centre Hospitalier Universitaire Sainte-Justine clinic) we found enrichment of CNVs affecting *GIT1* among cases (12 deletions and duplications, including 5 *de novo;* none in controls). The smallest focal deletion of 180 kb was found in an 11-year-old child (Fig. 5A, Table S7 case 6D) referred for developmental delay, with epilepsy and attention deficit hyperactivity disorder as comorbid conditions. Another focal deletion was found as a *de novo* event of 299 kb in a 10-year-old child referred for developmental delay (case 1D-DN). The DECIPHER database showed a similar *de novo* focal 282 kb deletion affecting a child referred for learning disabilities, dysphasia and poor motor coordination (see phenotype Table S7 case 5D-DN). This source also identified two *de novo* duplications affecting *GIT1*: a 1.4 Mb *de novo* duplication in a patient referred for intellectual disability, and a focal 466 kb duplication in a patient with broader developmental delay. A *de novo* damaging missense mutation was also reported in a schizophrenia case^47^. We conducted quantitative real-time PCR (rt-PCR) analysis to quantify relative mRNA expression (Supplementary text) of *GIT1* on 11 human tissues (including prenatal and adult brain) by targeting a critical exon of the gene. *GIT1* expression relative to that of the *MED13* gene (or of the *ACTB* gene) showed brain specific expression in prenatal and adult tissue (Fig. 5C).

### New candidate gene: *MVB12B*

**Figure 6:**
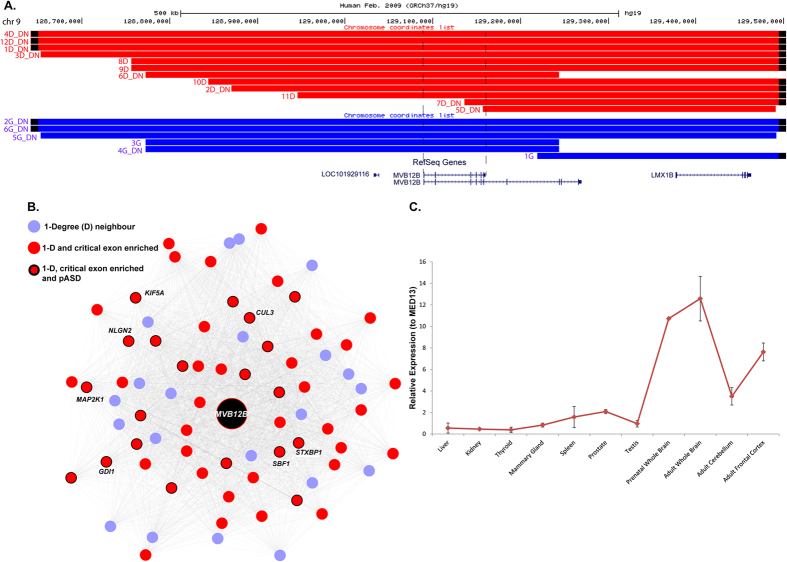
Deletions within *MVB12B* gene identified in developmental disorder cases and controls. (**A**) The breakpoints of 18 VOUS deletions (red) and duplications (blue) impacting *MVB12B* and nearby genes. The breakpoints include 12 *de novo* VOUS reported from developmental delay cases, including 3 recurrent breakpoints (6D-DN, 4G-DN, 3G). All *de novo* VOUS impacted the smaller isoform (highlighted by vertical dashed lines) of the gene (NM_001011703.2), and this was not impacted by CNV in controls. (**B**) The human protein co-expression network revealed that the *MBV12B* gene is the within the blue protein module and enriched for ‘critical exons’ (red nodes) and putative ASD genes reported to have *de novo* mutations (red node with black outline). (**C**) Expression of *MVB12B* (primer targeting critical exons) from quantitative real-time PCR (qRT-PCR) relative to housekeeping gene, *MED13* (replicated with another housekeeping gene *ACTB*) in 11 different tissues.

We ascertained another candidate gene, multivesicular body subunit 12B (*MVB12B*), with enrichment of critical exons, and also clustered with genes in the ‘blue’ network that were reported to be impacted by *de novo* exonic mutations in individuals with neuropsychiatric conditions. Initially we observed a single *de novo* duplication in our cohort, but subsequently found 18 VOUS involving this gene, including 12 that were *de novo* in origin (8 deletions and 4 duplications) (Fig. 6). This included 3 recurrent *de novo* CNVs ascertained from developmental delay cases impacting only the *MVB12B* gene (Fig. 6 and Table S7 cases 6D-DN,4G-DN and 3G). The protein encoded by this gene is an endosomal sorting complex required for transport (ESCRT-I)^48^ which is involved in sorting of ubiquitinated cargo protein from the plasma membrane. Mutations in the ESCRT complex family have been implicated in frontotemporal dementia, a neurodegenerative disease^49^. Our data included a *de novo* 839 kb duplication in a child (case 5G-DN) with Tourette syndrome, attention deficit hyperactivity disorder and learning disability. Another case had an adjacent *de novo* 700 kb duplication, and was described as having severe intellectual disability, whereas an individual (Fig. 6 case 3G) with a recurrent duplication was reported to be normal, thereby demonstrating variable expression (or reduced penetrance) of the duplication. Deletions were found in cases referred for developmental delay and reported to have other comorbid conditions (Table S7). The 73 kb smaller transcript of this gene (NM_001011703.2) containing the ‘critical exons’ in our analysis was impacted by 12 of our reported *de novo* VOUS and no CNVs in controls. Quantitative PCR analysis of 11 tissues for both *MVB12B* and *PPP1R9A* showed prenatal and adult brain-specific mRNA expression relative to that of the *MED13* gene (or the *ACTB* gene).

### New candidate gene: *PPP1R9A*

Our data set showed enrichment of deletion variants (4 VOUS, 1 *de novo*) containing another gene from our candidate list: the protein phosphatase 1 regulatory subunit 9A (*PPP1R9A*) gene (Fig. S7). Additional data sets revealed 18 CNVs (13 deletions and 5 duplications), including 3 additional *de novo* deletions impacting exons of the gene. *PPP1R9A* is the only gene from within the *de novo* variants that is enriched with critical exons and clustered within the blue protein module (Fig. S7B). The nearby genes are impacted by polymorphic deletions in controls, whereas no deletions encompassing *PPP1R9A* were identified from the control data set. A *de novo* missense mutation of this gene was recently reported in an ASD proband^40^. This gene shows tissue-specific imprinting; it is maternally expressed in skeletal muscle, but both alleles are expressed in other embryonic tissues, including the brain^50^. The protein encoded by this gene, Neurabin I, is a key candidate molecule in synaptic formation and function^50^. *PPP1R9A* also has a role in synaptic structure and function, spine motility and neurite formation^51^. We observed enrichment of critical exons of this gene within adult brain regions (Fig. S7) and it is part of the ‘blue’ module. An individual with autism was found to have a *de novo* missense variant of *PPP1R9A*^40^ and a rare LOF mutation was reported in a schizophrenia case^52^. This gene also shows increased expression in brain from individuals with bipolar disorder compared with controls^53^. Upon further investigation of the ‘blue’ protein co-expression network, highly connected neighboring (first degree) genes of *PPP1R9A* were reported to have *de novo* mutations in individuals with neuropsychiatric conditions^32^,^35^,^37^,^42^. In our cohort, a focal *de novo* 201 kb deletion affecting this gene was found in a child (Fig. S7 and Table S7 case 11D-DN) referred for language delay, repetitive behaviors and sensory sensitivities, consistent with her diagnosis of ASD. *A* 2 Mb deletion impacting 15 genes–including *PPP1R9A* and the sarcoglycan epsilon (*SGCE*) gene - was found in a 12 year old girl (case 7D) referred for myoclonus dystonia, short stature, failure to thrive, severe anxiety and obsessive-compulsive behavior. She also had mild dysmorphic features (triangular facies, broad forehead, thin lips) but no cognitive concerns were reported.

## Discussion

The brain transcriptome and proteomic method implemented here can infer candidate genes from within the boundaries of a pathogenic or VOUS CNV that are likely to impact brain-related conditions. Such lists of candidate genes can then be used to index the potential effect of a particular CNV or a set of CNVs in neurodevelopmental disorders.

The detection of genes critical for neurodevelopmental disorders is highly dependent on the breakpoints of the CNVs. Compared with sequencing technologies, clinical microarray tends to extend CNV breakpoints due to low probe density for the genome^54^. The low resolution impacts the precision of apparent breakpoints^55^, and CNVs may appear to encompass genes that may not be impacted by the real variant. Such false positives are reduced by large cohorts and high-resolution breakpoint detection through sequencing. Although HPM is one of the most comprehensive protein expression datasets, it may not be perfect in the quantification of peptides across all tissues partly due to the peptide fractionation techniques. Further improvement on fractionation technique will provide more concrete protein expression profile of genes across different tissue types.

We have demonstrated a quantifiable approach to screen for genes that are candidates to be involved in neurodevelopmental disorders, through the coordinated application of multiple genome-scale data sets. The critical exon approach reveals a negative selective pressure, whereas the protein expression analysis brings out networks that are in pathways biologically relevant to neurodevelopmental disorders^31^,^56^. This approach using CNVs could be augmented, through sequence analysis, to identify new genes, pathways and regulatory elements for a wide spectrum of neurodevelopmental phenotypes. Our recent work^57^,^58^ has shown that CNVs and smaller sequence-level variants (indels/single nucleotide variants (SNVs)) contribute - to autism spectrum disorder, and that whole genome sequencing, (which is capable of detecting both SNVs and CNVs) will become the ultimate standard for clinical genetic testing^35^,^59^. An approach such as ours that can assess the effects of mutations on genes in phenotypically relevant tissues will be important to reveal candidates for other classes of disorders. As large CNV and sequence level mutation datasets become available, large tissue specific RNA^60^ and protein expression dataset can be utilized for additional disease associations.

Through the approach described in this study, we have demonstrated the combined use of different types of molecular data from the human brain to interpret and identify candidate genes for developmental disorders, from pathogenic variants and VOUS. This quantifiable approach begins to enable the indexing of genes affected by CNVs for their potential role in neurodevelopmental disorders. Further functional characterization of these candidate genes and their products will allow us to define their regulation among tissues and throughout development. This, in turn, may aid in steps for timely interventions to mitigate untoward effects of various genomic alterations.

## Materials and Methods

### Clinical microarray datasets

The clinical microarray (CMA) data were obtained from two independent sites: The Hospital for Sick Children (SickKids) (7.106 cases) and Credit Valley Hospital (CVH) (3.513 cases) from individuals referred for investigation of developmental delay (Table S1). In both sites, a International Standards for Cytogenomic Arrays ISCA 180 K comparative genomic hybridization array (aCGH) was used to detect large CNVs by applying a circular binary segmentation algorithm^61^. For reference, we used a pool of 10 samples to compare individual probe intensities. The clinical annotation for each sample variant was conducted by the clinical laboratory geneticist in each site.

Briefly, DNA from each case and pooled same-sex reference DNA (Promega, Madison, WI) were differentially labeled with Cy3-dCTP or Cy5-dCTP, respectively, and hybridized to the array slide according to the manufacturer’s protocol (Oxford Gene Technology). We scanned arrays using the Agilent G2505Bmicroarray scanner and analyzed data using the Agilent Feature Extraction software (10.7.11) and CytoSure Interpret Software version 3.4.3 (OGT). Clinical interpretation of copy number variants was consistent with the American College of Genetics and Genomics guidelines^62^. When needed, we performed fluorescence *in situ* hybridization) analysis on cultured lymphocytes of parents, using standard protocols. Metaphase chromosomes were counter-stained with 4’,6-diamidino-2-phenylindole, and inverted grey scale imaging was used to visualize chromosome banding patterns for chromosome identification, using the ISIS Metasytems imaging software version 5.5.4 (Newton, MA, USA). Deletions of less than 200 kb and duplications less than 700 kb were followed up by aCGH in parental samples.

As controls, we used data from 9,692 unrelated samples from individuals with no obvious psychiatric history, from multiple major population-scale studies that used high-resolution microarray platforms. These included 4,347 control samples assayed by Illumina 1 M from the Study of Addiction Genetics and Environment (SAGE)^63^ and the Health, Aging, and Body Composition (HABC)^64^; 2,988 control samples assayed by Illumina Omni 2.5 M from the Collaborative Genetic Study of Nicotine Dependence (COGEND)]^65^ and Cooperative Health Research in the Region of Augsburg KORA projects^66^; 2,357 control samples assayed by Affymetrix 6.0 from the Ottawa Heart Institute^67^ and the PopGen project^68^. In addition, we incorporated 11,255 control datasets assayed on Illumina platforms from ARIC and Wellcome Trust case control consortium (WTCCC2) projects^6^.

### Critical exon classification

#### Burden of rare missense mutations

We used whole genome sequence data from the 1000 genomes project^25^ initiated by the US National Health Heart, Lung and Blood Institute (NHLBI) to calculate the burden of rare missense mutations in humans (495 males, and 544 females). Exonic regions had mean sequenced coverage of at least 20X. We used the RefSeq gene annotation model (which includes all exons from annotated isoforms) for our analysis. Genes with no variant calls were excluded. As described previously^24^, we annotated the variants using Annovar, and considered rare missense and LOF variants as strong proxies for recent (mostly within the last 5,000–10,000 years) rare deleterious mutation events in humans.

#### Spatio-temporal expression in human brain

We downloaded normalized RNA-seq data for spatio-temporal expression profiles of human brains from the BrainSpan database (http://www.brainspan.org/static/download.html). We analyzed 388 tissue samples from 32 post-mortem brain donors (prenatal and adult). The expression measures for exons were provided as reads per kilobase per million (RPKM) from mapped reads. Method details for sequencing, alignment, quality control and expression quantification can be found in the BrainSpan Technical White Paper (http://www.brainspan.org/). We conducted our spatio-temporal (prenatal and adult) analysis on 16 brain regions, including 11 neocortex regions (V1C, primary visual cortex; STC, posterior (caudal) superior temporal cortex; IPC, posterior inferior parietal cortex; A1C, primary auditory cortex; S1C, primary somatosensory cortex; M1C, primary motor cortex; DFC, dorsolateral prefrontal cortex; MFC, medial prefrontal cortex; VFC, ventrolateral prefrontal cortex; OFC, orbital frontal cortex; ITC, inferolateral temporal cortex) and AMY, amygdaloid complex; CBC, cerebellar cortex; HIP, hippocampus; MD, mediodorsal nucleus of thalamus; and STR, striatum. We classified critical exons as described previously^24^.

### Data from human protein expression at developmental stages

We used high-resolution genome-wide Fourier-transform mass spectrometry data (downloaded from the Human Proteome Map)^19^ to analyze protein expression levels in human tissues at two developmental stages. This included in-depth proteomic profiling of 30 histologically normal human samples, including 17 adult tissues (lung, heart, liver, gall bladder, adrenal gland, kidney, urinary bladder, prostate, testis, ovary, rectum, colon, pancreas, oesophagus, retina, frontal cortex, and spinal cord) and 7 fetal tissues (liver, heart, brain, placenta, gut, ovary, testis)^19^. High-resolution Fourier transform mass spectrometers had been used for fragmentation (high-high mode) to process the data. This resulted in the identification of proteins encoded by 17,294 genes, accounting for approximately 84% of annotated protein-coding human genes^19^. We used average spectral counts per gene per sample as the measure for protein expression.

### Weighted gene coexpression network analysis (WGCNA)

We used the R WGCNA package^69^,^70^ to analyze the human protein expression. The use of weighted networks represents an improvement over unweighted networks because it preserves continuity of the co-expression information, and it is biologically robust with respect to parameter ß^33^. We excluded proteins that are rarely expressed (expression = 0 in at least 90% of the samples) because such low-expressed features tend to reflect noise, and correlations based on such counts are not really meaningful. We calculated the absolute value of the Pearson correlation coefficient for all pair-wise comparisons of protein expression values across all developmental tissue samples into a similarity matrix. We used blockwise network construction and a module detection method, where a block cluster consists of a maximum of 20,000 proteins. We constructed a signed adjacency matrix using a “soft” power adjacency function a_ij_ = |0.5 + 0.5 * cor(x_i_, x_j_)|^β^ , where the absolute value of the Pearson correlation measures protein co-expression similarity, and a_ij_ represents the resulting adjacency–reflecting the connection strength. We chose for our analysis the soft threshold beta = 18, based on the scale-free topology^33^ criterion ß (63). Next, to compute modules, where the proteins have high “topological overlap”, we compared connection strength between proteins in the network. The parameters for module detection were: minimum 30 proteins per module and a medium sensitivity deepsplit = 2 was applied to cluster splitting. The clustering of genes for modules used average linkage hierarchical clustering; modules were identified in the resulting dendrogram by the dynamic hybrid tree cut. Such modules were trimmed of genes whose correlation with module eigengene (KME) was less than a threshold defined by the function minKMEtoStay; for merging similar modules, we used 0.35 as a threshold. The connectivity of each node i is the sum of connections to other nodes.

For visualizing the protein co-expression network, we used Cytoscape network software v.2.8.3. A node is represented by a circle, and the edge represented by a line between the nodes implies the co-expression weighted Pearson distance. The color of the node represents membership to a phenotype.

### Significant test analysis and permutation test

We used Fisher’s exact test (FET) for all count data and g p-value <0.05 (after Bonferroni multiple test correction) as the threshold for significance in tests of gene enrichment. To reveal the strength of enrichment association with the gene lists, we undertook a permutation test by randomly drawing equal numbers of genes and re-analyzing the data under the null-hypothesis. The random draw was conducted from a background appropriate for the test. To analyze enrichment of critical exon genes (top 25^th^ percentile) within the ‘blue’ protein module (Fig. 4D), the background included all genes for which we had both protein expression and mRNA expression from RNA-seq data. For the analysis of pathogenic and VOUS deletion/duplication gene enrichment within the ‘blue’ protein module, we used all genes with protein expression as the background. In each iteration of the random draw, an equal (to the original set) number of genes was drawn. With sufficient iterations (100,000 times), the resulting sets of p-values are presumed to be a reasonable approximation of the null distribution of the p-values.

### Reverse transcription polymerase chain reaction (RT-PCR) and quantitative RT-PCR (qRT-PCR)

To quantitate ‘critical exons’ by qRT-PCR, primers were designed to prime from within the specific exon (Supplementary Table S8). We tested PCR efficiency with a dilution standard curve, and for specificity with melting curve analysis using adult whole brain cDNA. To quantify the ‘critical exon’ expression from selected genes, we used RNA from a panel of 11 human tissues: liver (BD Biosciences), kidney (Stratagene), mammary gland (BD Biosciences), cerebellum (Clonetech), skeletal muscle (Stratagene), prostate (Clonetech), spleen (Stratagene), thyroid (Stratagene) and testis (Clonetech). Reverse transcription was performed using the Superscript III First strand Synthesis Supermix (Invitrogen). We used 10 ng of cDNA as template for RT-PCR under standard PCR conditions, using Brilliant III SYBR^®^ Green PCR Master Mix (Agilent) and the MX300 software (Agilent). Gene expression was normalized using *MED13* or *ACTB* (dCt) and quantified as relative expression (2^(-dCt)).

## Additional Information

**Accession codes**: The dataset used for the analyses described in this manuscript was obtained from the database of Genotype and Phenotype (dbGaP) found at Accession Number: phs001154.v1.p1.

**How to cite this article**: Uddin, M. *et al.* Indexing Effects of Copy Number Variation on Genes Involved in Developmental Delay. *Sci. Rep.* **6**, 28663; doi: 10.1038/srep28663 (2016).

## Supplementary Material

Supplementary Information

Supplementary Tables
