# Supplementary material for: Indexing Effects of Copy Number Variation on Genes Involved in Developmental Delay
Source: Sci Rep. 2016 Jul 1;6:28663. doi: 10.1038/srep28663 (PMC4929460; doi:10.1038/srep28663)
Supplement: Supplementary Information [file srep28663-s1.pdf]

Indexing Effects of Copy Number Variation on Genes Involved in Developmental Delay

(Supplementary Material)

Mohammed Uddin[1,2], Giovanna Pellecchia[1,2], Bhooma Thiruvahindrapuram[1,2], Lia D'Abate[1,2,3], Daniele Merico[1,2], Ada Chan[1,2,3], Mehdi Zarrei[1,2], Kristiina Tammimies[4], Susan Walker[1,2], Matthew J Gazzellone[1,2], Thomas Nalpathamkalam[1,2], Ryan KC Yuen[1,2], Koenraad Devriendt[5], Géraldine Mathonnet[6], Emmanuelle Lemyre[6], Sonia Nizard[6], Mary Shago[7], Ann M. Joseph-George[7], Abdul Noor[8], Melissa T Carter[9], Grace Yoon[10], Peter Kannu[10], Frédérique Tihy[6], Erik C. Thorland[11], Christian R Marshall[1,7], Janet A. Buchanan[1,2], Marsha Speevak[12], Dimitri J Stavropoulos[7], Stephen W Scherer[1,2,13,3]

1) The Centre for Applied Genomics, The Hospital for Sick Children, Toronto, Ontario, Canada; 2) Program in Genetics and Genome Biology (GGB), The Hospital for Sick Children, Toronto, Ontario, Canada; 3) Department of Molecular Genetics, University of Toronto, Toronto, Ontario, Canada. 4) Center of Neurodevelopmental Disorders (KIND), Neuropsychiatric Unit, Department of Women's and Children's Health, Karolinska Institutet, Stockholm, Sweden; 5) Center for Human Genetics, University of Leuven, Leuven, Belgium 6) CHU Sainte-Justine, University de Montreal, Montreal, Quebec, Canada. 7) Genome Diagnostics, Pediatric Laboratory Medicine, The Hospital for Sick Children, Toronto, Ontario, Canada; 8) Department of Pathology and Laboratory Medicine, Division of Diagnostic Medical Genetics, Mount Sinai Hospital, Toronto, Ontario, Canada; 9) Department of Genetics, The Children's Hospital of Eastern Ontario, Ottawa, ON, Canada; 10) Division of Clinical and Metabolic Genetics, Department of Pediatrics, The Hospital for Sick Children, University of Toronto, Toronto, Ontario M5G 2L3, Canada; 11) Cytogenetics Laboratory, Department of Laboratory Medicine and Pathology, Mayo Clinic, Rochester, Minnesota; 12) Department of Laboratory Medicine and Pathobiology, University of Toronto, Toronto, Ontario, Canada; 13) McLaughlin Centre, University of Toronto, Toronto, Ontario, Canada;

Clinical Microarray Datasets

The clinical microarray (CMA) data was obtained from two independent sites, The Hospital for Sick Children (SickKids) and Credit Valley Hospital (CVH). A total of 7,106 and 3,513 cases CMA data were obtained, respectively, who went through confirmed diagnosis for DD (Table S1). In both sites, ISCA 180K comparative genomic hybridization array was used to detect large CNVs by applying circular binary segmentation algorithm. For reference, we used a pool of 10 samples to compare individual probe intensities. The clinical annotation for each sample variant was conducted by the clinical geneticist in each site.

DNA extracted from peripheral blood was used to perform comparative genomic hybridization array (aCGH) analysis on a custom designed 4 X 180K oligonucleotide microarray platform (Oxford Gene Technology (OGT), Oxford, UK). Microarray experiments were performed according to the manufacturer's instructions. Briefly, DNA from the proband and pooled same-sex reference DNA (Promega, Madison, WI) were labeled with Cy3-dCTP and Cy5-dCTP, respectively and were hybridized to the array slide according to the manufacturer's protocol (OGT). The arrays were scanned using the Agilent G2505Bmicroarray scanner. Data analysis was performed using the Agilent Feature Extraction software (10.7.11) and CytoSure Interpret Software version 3.4.3 (OGT). Clinical interpretation of copy number variants was consistent with the ACMG guidelines[1]. Parental follow-up studies were performed by FISH analysis on cultured lymphocytes using standard protocols. Metaphase chromosomes were counter-stained with DAPI, and inverted grey scale imaging was used to visualize chromosome banding patterns for chromosome identification, using the ISIS Metasytems imaging software version 5.5.4 (Newton, MA, USA). Parental follow-up of deletions less than 200 kb and duplications less than 700 kb were performed by aCGH.

We have used 9,692 unrelated control samples from multiple major population scale studies that used high-resolution microarray platform (Table S3). These samples do not have any obvious psychiatric history. The studies include 4,347 control samples assayed in Illumina 1M from the Study of Addiction Genetics and Environment (SAGE)[2] and the Health, Aging, and Body Composition (HABC)[3]; 2,988 control samples assayed in Illumina Omni 2.5M from COGEND[4] and KORA projects[5]; 2,357 control samples assayed in Affymetrix 6.0 from Ottawa Heart Institute (OHI)[6] and PopGen project[7]. In addition, we have incorporated additional 11,255 control datasets assayed in Illumina platforms from ARIC and WTCC2 project[8].

1. Critical Exon Classification

For critical exon classification described in (Uddin et al, 2014)[9], we used the 1000 genomes project for rare missense loss of function (LOF) mutation burden computation and transcriptome data from the human developmental brain atlas.

a. Burden of rare missense mutations

We used data from the 1000 genomes project[10] initiated by the US National Health Heart, Lung and Blood Institute (NHLBI) to calculate the burden of rare missense mutations in human populations. 1,039 whole genome sequencing samples (495 males, and 544 females)[10]. Within these whole genome sequenced (WGS) samples, exonic regions had mean coverage of at least 20X. We used the RefSeq gene annotation model (which includes all exons from annotated isoforms) for our analysis. Genes with no variant calls were excluded. As described previously[9], we annotated the variants using Annovar and considered rare missense and loss of function (LOF) variants as strong proxy for recent (mostly within the last 5,000-10,000 years) rare deleterious mutation events in humans.

b. Spatio-temporal Human Brain Expression:

The normalized RNA-seq expression profiles of spatio-temporal developmental human brains were downloaded from the BrainSpan database (http://www.brainspan.org/static/download.html). We have analyzed 388 tissue samples from 32 post mortem donors (prenatal and adult). The expression measures were provided for exons as reads per kilobase (kb) per million (RPKM) from mapped reads. Method details for sequencing, alignment, QC and expression quantification can be found in the BrainSpan Technical White Paper (http://www.brainspan.org/). We have conducted our spatial-temporal (prenatal and adult) analysis on 16 brain regions, including 11 neocortex regions (V1C, primary visual cortex; STC, posterior (caudal) superior temporal cortex; IPC, posterior inferior parietal cortex; A1C, primary auditory cortex; S1C, primary somatosensory cortex; M1C, primary motor cortex; DFC, dorsolateral prefrontal cortex; MFC, medial prefrontal cortex; VFC, ventrolateral prefrontal cortex; OFC, orbital frontal cortex; ITC, inferolateral temporal cortex) and AMY, amygdaloid complex; CBC, cerebellar cortex; HIP, hippocampus; MD, mediodorsal nucleus of thalamus; and STR, striatum. To classify critical exon, we have computed the 75th percentile[9] value from the entire dataset and used it as a threshold to define exons with high and low expression. Critical exon fraction was computed for a gene or a group of genes

(impacted by CNVs) by applying the 75th percentile index on all exons. The fraction was computed by dividing the number of exons classified as critical exon over total number of exons.

2. Human Developmental Protein Expression Data

The protein expression levels for the genes were analyzed using high-resolution genome-wide Fourier-transform mass spectrometry data[11] (downloaded from Human Proteome Map). We have used in-depth proteomic profiling of 30 histologically normal human samples, including 17 adult tissues (lung, heart, liver, gall bladder, adrenal gland, kidney, urinary bladder, prostate, testis, ovary, rectum, colon, pancreas, oesophagus, retina, frontal cortex, and spinal cord) and 7 fetal tissues (liver, heart, brain, placenta, gut, ovary, testis)[11]. High-resolution Fourier transform mass spectrometers used for fragmentation (high-high mode) to process the data. The data resulted in the identification of proteins encoded by 17,294 genes accounting for approximately 84% of the total annotated protein-coding genes in humans[11]. Average spectral counts per gene per sample were used as the measure for protein expression.

3. WGCNA Network details:

We have used weighted coexpression network analysis (WGCNA) analysis using human protein expression data in development. The R WGCNA package was used to conduct the analysis[12,13]. The use of weighted networks represents an improvement over unweighted networks because it preserves continuous nature of the co-expression information and it is biologically robust with respect to parameter ß[14]. We excluded proteins that are not expressed (expression = 0) in at least 90% of the samples because such low-expressed features tend to reflect noise and correlations based on counts that are mostly zero are not really meaningful. The absolute value of the Pearson correlation coefficient is calculated for all pair-wise comparisons of protein expression values across all developmental tissue samples into a similarity matrix. We used blockwise network construction and module detection method where the clustering of a block will consists maximum of 20,000 proteins. A signed adjacency matrix was constructed using a “soft” power adjacency function $a_{ij} = |0.5 + 0.5 * cor(x_i, x_j)|^{\beta}$ where the absolute value of the Pearson correlation measures protein the co-expression similarity, and a_{ij} represents the resulting adjacency that measures the connection strengths. We have chosen the soft thresholding beta = 18 based on the scale-free topology[14] criterion ß for our analysis. Next, to compute modules, where the proteins have high “topological overlap”, we compared connection strength between proteins in the network. The parameters for module detection used –were: minimum 30 proteins per module and

a medium sensitivity deepsplit = 2 was applied to cluster splitting. The clustering of genes for modules used average linkage hierarchical clustering and modules are identified in the resulting dendrogram by the dynamic hybrid tree cut. Found modules are trimmed of genes whose correlation with module eigengene (KME) is less than a threshold defined by the function minKMEtoStay and for merging similar modules, we used 0.35 as a threshold. The connectivity of each node i is the sum of connections to other nodes.

For visualizing the protein co-expression network, Cytoscape network software v.2.8.3 was used. The node are represented by a circle and the edge between the nodes implies the co-expression weighted Pearson distance. The color of the node is representative of their membership to a phenotype.

4. Significant Test Analysis and Permutation Test

We have used Fisher's exact test (FET) for all count data and gene enrichment test with p-value < 0.05 (after Bonferroni multiple test correction) as the threshold for significance. To reveal the strength of enrichment association with the gene lists, we undertook a permutation test by randomly drawing equal numbers of genes and re-analyzing the data under the null-hypothesis. The random draw was conducted from a background that is appropriate for the test. With sufficient iterations (100,000 times), the resulting sets of *p*-values are presumed to be a reasonable approximation of the null distribution of the *p*-values.

5. Reverse Transcription Polymerase Chain Reaction (RT-PCR) and quantitative RT-PCR (qRT-PCR)

For the quantification of 'critical exons' by qRT-PCR, primers were designed to prime from within the specific exon (Supplementary **Table S8**). The primers were tested for their PCR efficiency by dilution standard curve and for specificity with melting curve analysis using adult whole brain cDNA. To quantify the 'critical exon' expression from selected genes, we used RNA from a panel of 11 human tissues: liver (BD Biosciences), kidney (Stratagene), mammary gland (BD Biosciences), cerebellum (Clonetech), skeletal muscle (Stratagene), prostate (Clonetech), spleen (Stratagene), thyroid (Stratagene) and testis (Clonetech). Reverse transcription was performed using the Superscript III First strand Synthesis Supermix (Invitrogen). Using 10ng of cDNA as template, RT-PCR was performed under standard PCR conditionsusing Brilliant III SYBR® Green PCR Master Mix (Agilent) and the MX300 software (Agilent). Gene expression was normalized using *MED13* or *ACTB* (dCt) and quantified as relative expression (2^(-dCt)).

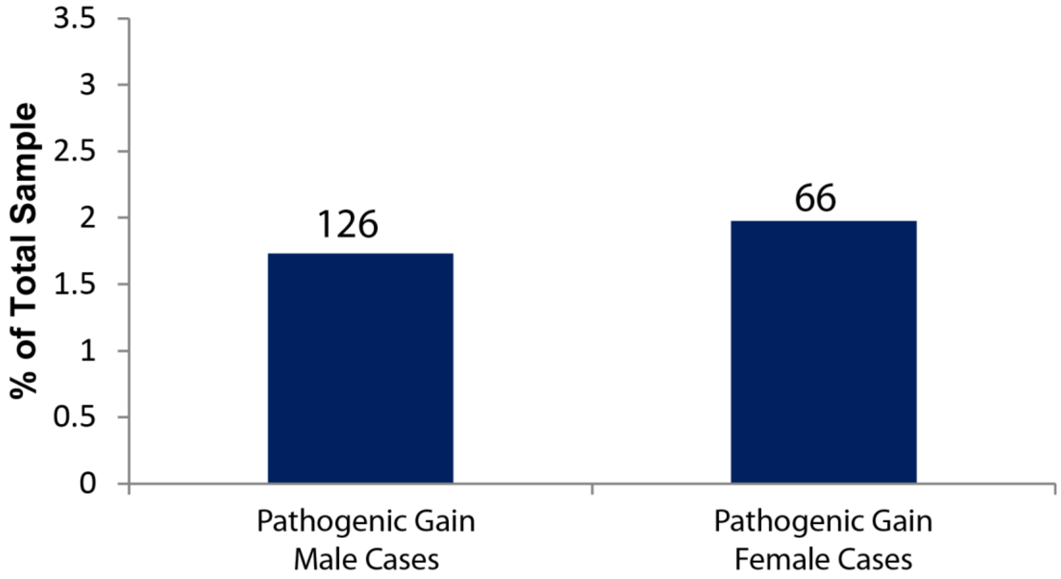

Figure S1. Percentage of pathogenic gain in males and females. The percentages of male and female carrying pathogenic duplication variants in our dataset.

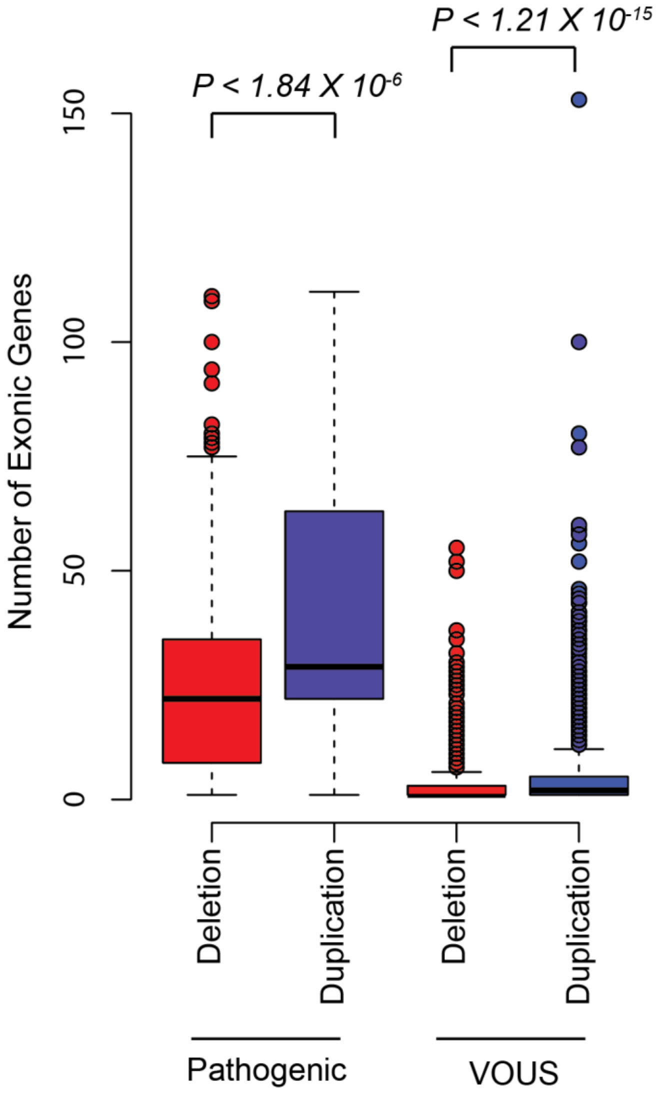

Figure S2. Distribution of exonic genes. The distribution of exonic genes impacted by pathogenic and VOUS deletion (red) and duplication (blue) in our developmental delay dataset.

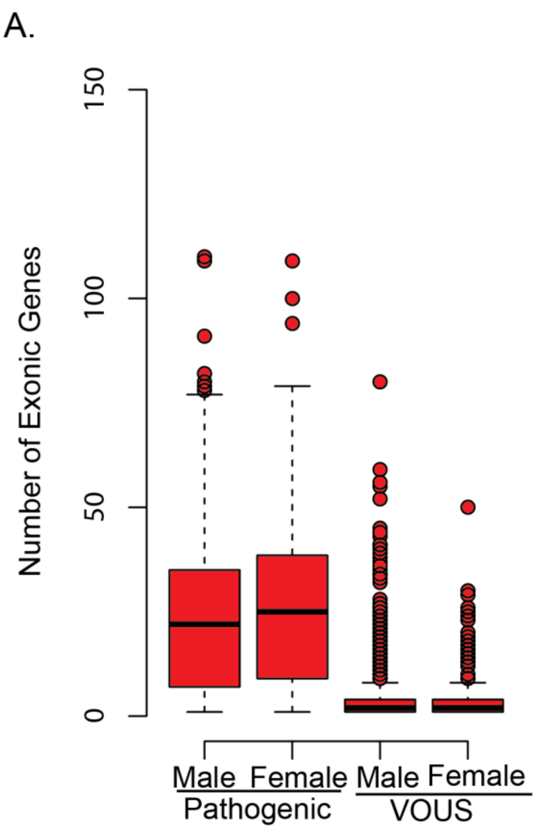

Figure S3. Gene distribution of male-female variants. The number of exonic genes impacted by (A) deletion and (B) duplication variants in male and females.

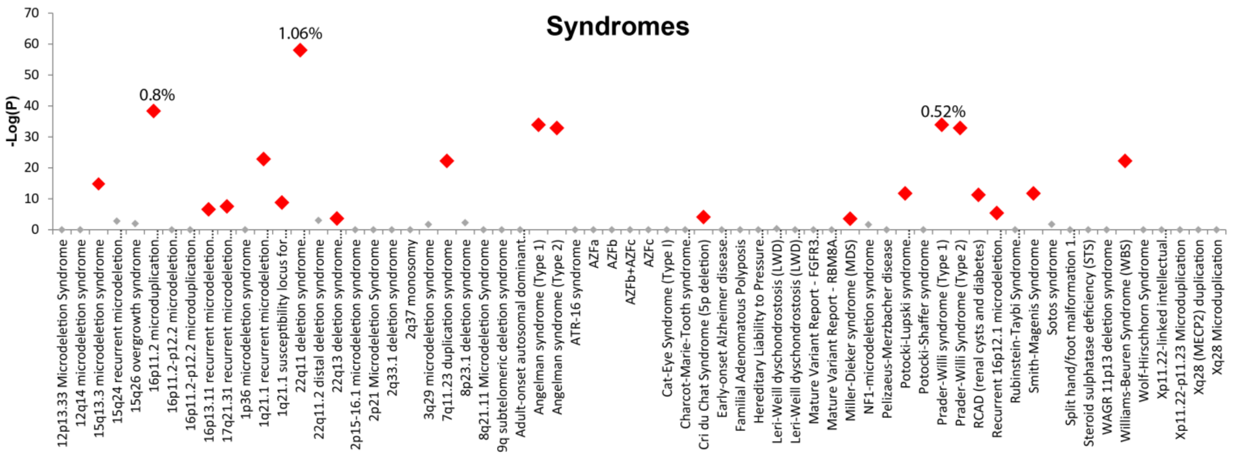

Figure S4. DECIPHER syndromes enrichment in DD dataset. We observed pathogenic variants of 0.86% frequency in 16p11.2, 1.06% in 22q11, 0.52% Angelman syndrome and Prader-Willi syndrome.

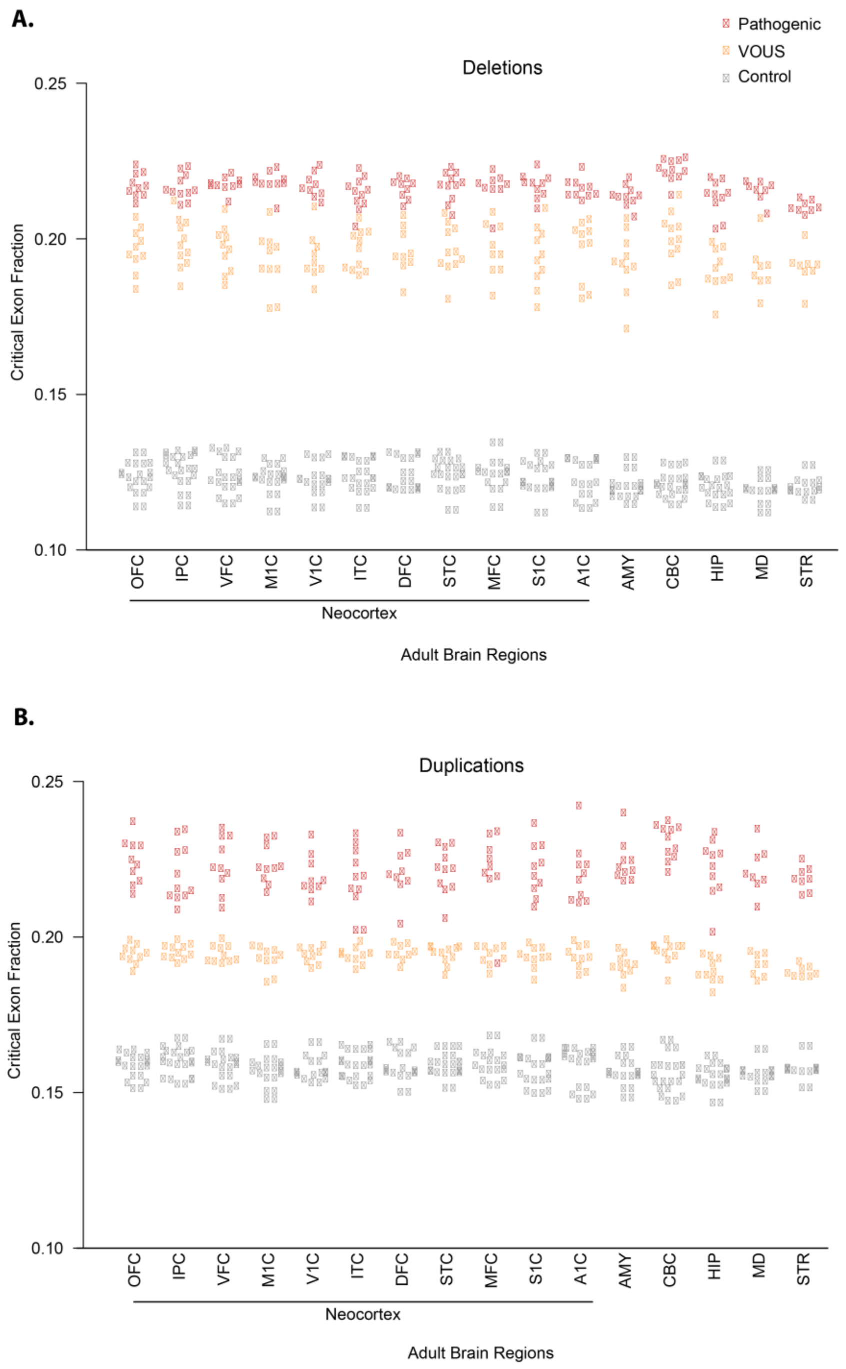

Figure S5. The fraction of critical exons (over all exons) is computed from human prenatal brain regions for the genes impacted by pathogenic, VOUS and rare control deletion and duplication variants. The critical exon fraction was computed using gene expression level quantified from RNA sequencing in 388 brain tissues (controls) from 32 postmortem donors in 2 developmental periods (prenatal and adult) for 16 brain regions (AMY, amygdaloid complex; CBC, cerebellar cortex; V1C, primary visual cortex; STC, posterior (caudal) superior temporal cortex; IPC, posterior inferior

parietal cortex; A1C, primary auditory cortex; S1C, primary somatosensory cortex; M1C, primary motor cortex; STR, striatum; DFC, dorsolateral prefrontal cortex; MFC, medial prefrontal cortex; VFC, ventrolateral prefrontal cortex; OFC, orbital frontal cortex; MD, mediodorsal nucleus of thalamus; ITC, inferolateral temporal cortex; HIP, hippocampus). The critical exon fraction computed using prenatal brain transcriptome is shown for the genes impacted by pathogenic (red dots) and VOUS (orange dots) (A) deletions and (B) duplications in comparison to genes impacted by rare controls deletions (gray dots).

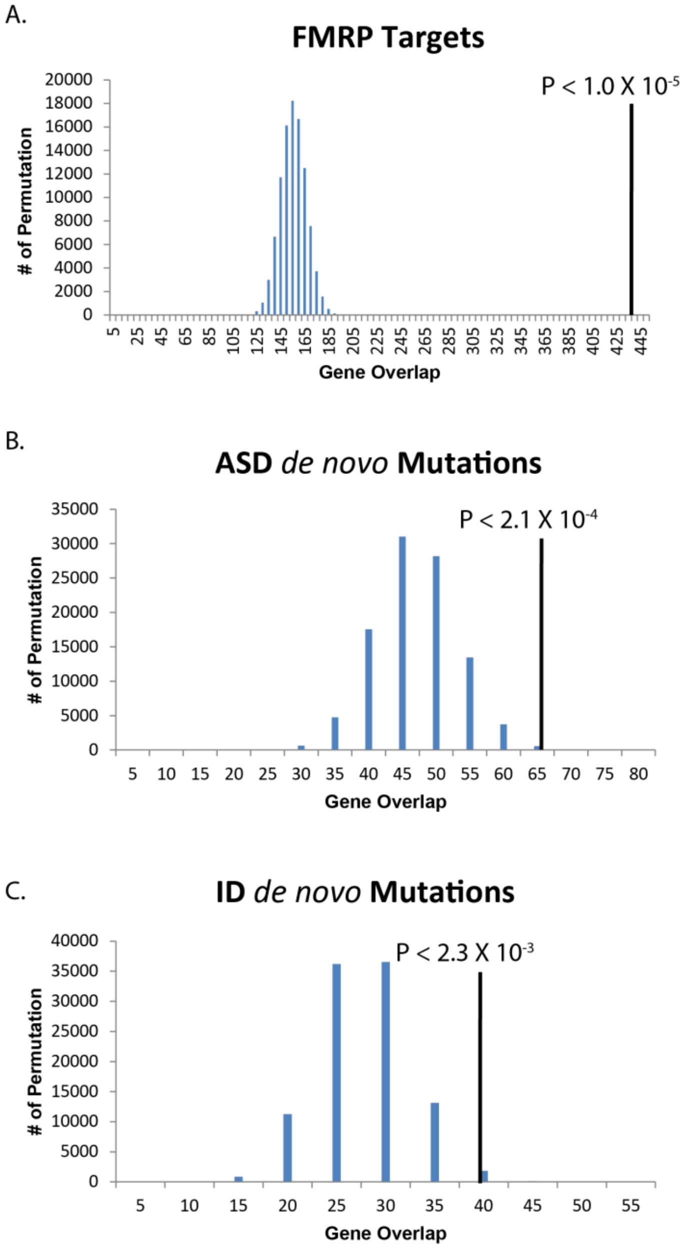

Figure S6. Gene enrichment analysis. In blue protein module, enrichment of (A) fragile syndrome FMR1 protien targets, (B) *de novo* mutations in autism spectrum

disorder, and (C) de novo mutations in intellectual disability was quantified after 100,000 random permutations.

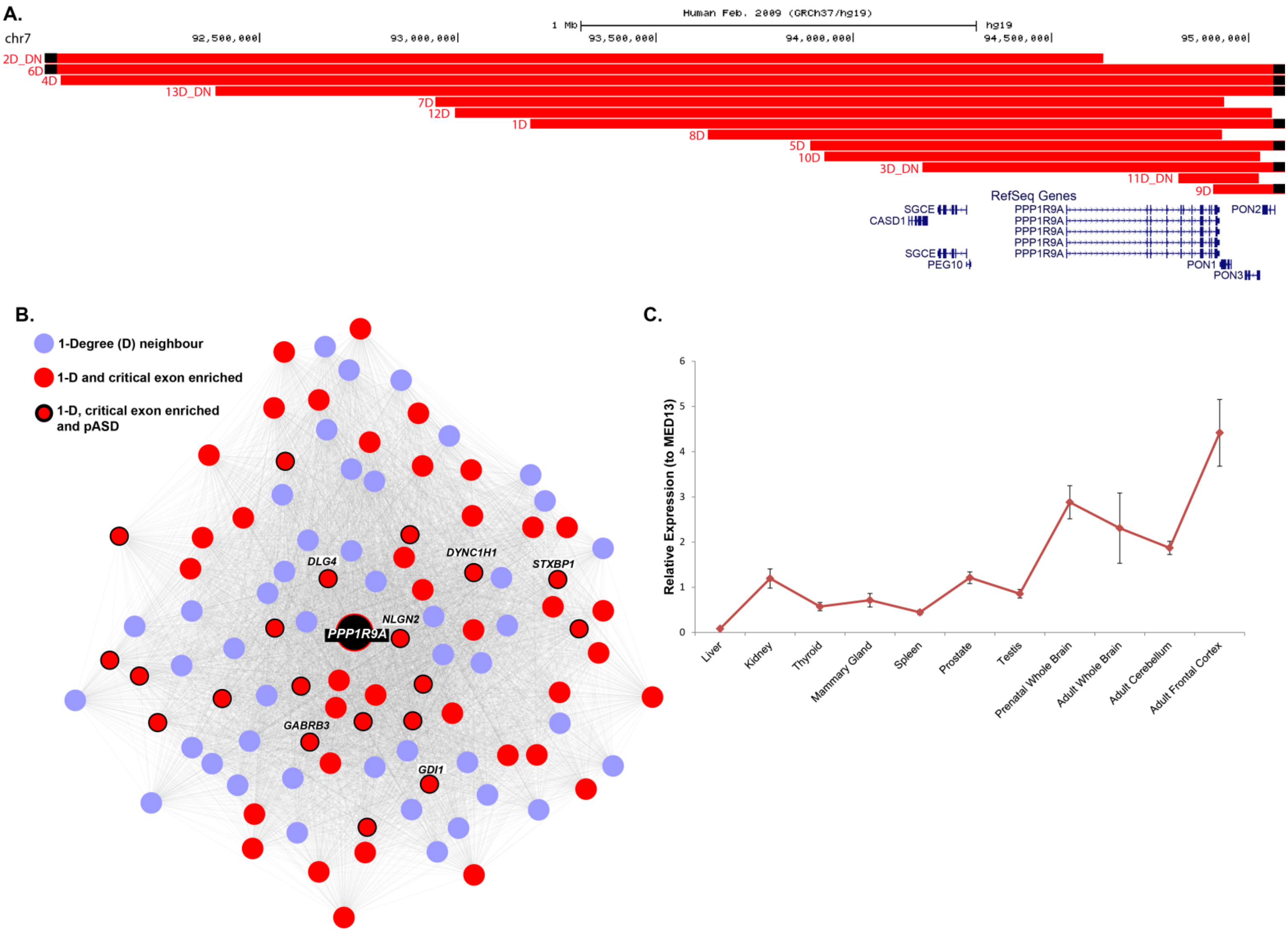

Figure S7. Deletions within *PPP1R9A* gene identified in developmental disorder cases and controls. (A) The breakpoints of 13 VOUS deletions (red) impacting *PPP1R9A* and nearby genes. The breakpoints include 4 *de novo* VOUS reported from developmental delay cases. There was no deletion found in our control dataset. The shortest *de novo* deletion is 201Kb ascertained from a case (11D_DN) with developmental delay in our cohort. This particular de novo also impacts PON gene family where exonic deletions also present in controls. (B) The human protein co-expression network revealed *PPP1R9A* gene is the within the blue protein module and enriched for 'critical exons' (red nodes) and putative ASD genes reported to have de novo mutations (red node with black outline). (C) Expression of *PPP1R9A* (primer targeting critical exons) from quantitative real-time PCR (qRT-PCR) relative to

housekeeping gene, *MED13* (replicated with another housekeeping gene *ACTB*) in 11 different tissues.

Table S1. Developmental delay cohort.

Data	Broad phenotype	Female	Male	Total
SK	Developmental Delay	2245	4861	7106
CVH	Developmental Delay	1096	2417	3513
**Total**		3341	7278	10619
Bradly Coe (Bradly Coe et al. 2014)	Developmental Delay	7076	10282	17358
**Total**		10417	17560	27977
SK	Congenital Abnormalities	864	957	1821
**Total**		11281	18517	**29798**

Table S2. Control cohort.

Dataset	Status	Male	Female	Total
Cogend_kora	Controls	1635	1353	2988
Habc_sage	Controls	2552	1795	4347
OHI_PopGen	Controls	1148	1209	2357
**Total**		5335	4357	9692
ARIC and WTCC2 Controls (Bradly Coe et al. 2014)	Controls			11255

Total				20947

Table S3. CNV length Distribution (30Kb-5Mb) for each case and control dataset (excel file provided as supplementary tables).

Table S4. Gene set enrichment analysis for each module. From the association analysis of 18,826 geneset, the most significant (Bonferroni Corrected) top 20 gene set is listed for each module (excel file provided as supplementary tables).

Table S5. Candidate genes from 'critical exon' and protein co-expression analysis. The annotation of each includes critical exon from prenatal and adult brain tissues. Also the genes ascertained in CS and VOUS in our DD dataset (excel file provided as supplementary tables).

Table S6. Clinically relevant genes within known syndromic regions.

Syndrome	Coordinate	Total Gene	Genes	Blue Protein Network and Critical Exon Enriched Genes
16p11.2 microduplication syndrome	chr16:29606852-30199855	30	*DOC2A,ASPHD1,LOC440356,CORO1A,TBX6,LOC100271831,PRRT2,CDIPT,QPRT,YPEL3,SLC7A5P1,PPP4C,MAPK3,SPN,MVP,FAM57B,ZG16,ALDOA,INO80E,SEZ6L2,TAOK2,KCTD13,MAZ,KIF22,GDPD3,C16orf92,C16orf53,TMEM219,C16orf54,HIRIP3*	*DOC2A,TAOK2,PRRT2,SEZ6L1,MAPK3,ALDOA*
Angelman syndrome (Type 1/2)	chr15:23619912-28438266	116	*NIPA2,NIPA1,SNORD116-9,SNORD116-8,SNORD116-5,SNORD116-4,SNORD116-7,SNORD116-6,SNORD116-1,SNORD116-3,SNORD116-2,SNORD109A,SNORD109B,GOLGA8IP,PARSN,PWRN1,PWRN2,OCA2,LOC100128714,MIR4508,SNORD115-34,PAR5,PAR4,IPW,PAR1,GOLGA8E,SNORD116-19,SNORD116-18,GABRG3,SNORD115-3,SNORD115-*	*UBE3A,GABRB3,CYFIP1*

			31,SNORD64,SNORD115-18,SNORD115-19,SNORD115-14,SNORD115-15,SNORD115-16,SNORD115-17,SNORD115-10,SNORD115-11,SNORD115-12,SNORD115-13,UBE3A,MAGEL2,ATP10A,C15orf2,SNORD115-21,LOC283683,SNORD115-23,SNORD115-22,SNORD115-25,SNORD115-8,SNORD107,SNORD115-26,SNORD115-29,SNORD108,SNORD116-16,SNORD115-20,HERC2,SNORD115-24,SNORD115-27,SNORD115-36,SNORD115-37,MKRN3,SNORD115-35,SNORD115-32,SNORD115-33,SNORD115-30,SNORD115-28,GABRA5,MIR4715,SNORD115-38,SNORD115-39,HERC2P2,HERC2P7,NDN,LOC503519,GABRB3,SNORD115-43,SNORD115-42,SNORD115-41,SNORD115-40,SNORD115-47,SNORD115-45,SNORD115-44,SNORD115-48,TUBGCP5,CYFIP1,SNORD116-24,SNORD116-25,SNORD116-26,SNORD116-27,SNORD116-20,SNORD116-21,SNORD116-22,SNORD116-23,SNORD116-28,SNORD116-29,SNORD115-6,SNORD115-7,SNORD115-4,SNORD115-5,SNORD115-2,SNURF,SNORD115-1,SNORD116-11,SNORD116-10,SNORD116-13,SNORD116-12,SNORD116-15,SNORD116-14,SNORD116-17,SNORD115-9,SNRPN,WHAMMP3,LOC653061	
Williams-Beuren	chr7:72744	26	*STX1A,WBSCR27,WBSCR22,LAT2,LIMK1,WBSCR28,MIR4284,R*	*CLIP2,LIMK1*

Syndrome (WBS) and 7q11.23 duplication syndrome	455-74142672		*FC2,FKBP6,MIR590,FZD9,VPS37D,ABHD11,CLIP2,CLDN3,CLDN4,BCL7B,ELN,MLXIPL,DNAJC30,GTF2IRD1,BAZ1B,TBL2,EIF4H,GTF2I,ABHD11-AS1*	
22q11 Velocardiofacial/DiGeorge syndrome	chr22:19009792-21452445	62	*P2RX6P,RIMBP3,TMEM191A,PI4KA,KLHL22,SLC7A4,LOC388849,MIR185,GNB1L,TBX1,MIR3618,MIR1306,SEPT5,ZNF74,P2RX6,DGCR8,PI4KAP1,DGCR10,TMEM191B,DGCR2,GP1BB,LOC400891,C22orf39,C22orf25,DGCR6L,MED15,CRKL,TXNRD2,CLDN5,LOC150197,RTN4R,TSSK2,GSC2,ARVCF,SLC25A1,MIR4761,COMT,LOC284865,LOC729444,AIFM3,CLTCL1,SERPIND1,THAP7-AS1,SCARF2,HIRA,THAP7,MIR1286,RANBP1,POM121L4P,SNAP29,UFD1L,DGCR11,C22orf29,MRPL40,DGCR14,ZDHHC8,CDC45,TRMT2A,LZTR1,LOC150185,MGC16703,SEPT5-GP1BB*	*CLDN5,CLTCL1,SEPT5* $^{\Psi}$*,PI4K2*
3q29 micro-deletion/duplication syndrome	chr3:195726835-197344663	28	*RNF168,NCBP2,LOC100507086,ZDHHC19,DLG1,TM4SF19-TCTEX1D2,TFRC,LOC152217,UBXN7,FBXO45,MIR4797,MFI2,SENP5,OSTalpha,TCTEX1D2,PIGX,PIGZ,LOC220729,BDH1,PCYT1A,WDR53,LRRC33,MFI2-AS1,C3orf43,LOC401109,TM4SF19,CEP19,PAK2*	*PAK2* $^{\Psi}$

Ψ – deleterious point mutations or focal deletions have been independently reported in cases with developmental delay or related conditions.

Table S7. Phenotypic table for cases with developmental delay and CNVs impacting *GIT1*, *PPP1R9A*, and *MVB12B* gene. The cases are listed only if the phenotypic information was available.

Case ID	Critical Exon Gene	Size	Copy Number	CNV (Inheritance)	Age of Ascertainment	Developmental Delay/ ID	Dysmorphic Features	Other Clinical Features
1D_DN	*GIT1*	299 Kb	Loss	17q11.2 27.822 to 28.121 Mb (*de novo*)	10 yrs	Developmental delay	N/A	
2D	*GIT1*	3.1 Mb	Loss	27.869 to 31.043 Mb	N/A	Developmental delay and/or ID	N/A	N/A
3D	*GIT1*	2.1 Mb	Loss	27.606 to 29.722 Mb	N/A	Developmental delay and/or ID	N/A	N/A
3G_DN	*GIT1*	466 kb	Gain	17q11.2 27.696 to 28.162 Mb (*de novo*)	< 1 yr	N/A	N/A	
4D_DN	*GIT1*	5.3 Mb	Loss	17q11.2q12 27.771 to 33.094 Mb (*de novo*)	1 yr	N/A	N/A	
5D_DN	*GIT1*	282 Kb	Loss	17q11.2 27.837 to 28.120 Mb (*de novo*)	N/A	Learning disability		Dysphasia
5G	*GIT1*	9.2 Mb	Gain	17p11.2q12 20.649 to 29.832	14 yr	ID		Autism, obesity
6D	*GIT1*	180 Kb	Loss	17q11.2 27.733 to 27.913 Mb	11 yrs	Developmental delay		Epilepsy, ADHD
7D_DN	*GIT1*	3.5 Mb	Loss	17q11.2 27.274 to 30.817 Mb (*de novo*)	3 months	-	-	Prematurity, tetralogy of Fallot, bilateral choroid plexus cyst, imperforate anus, ambiguous genitalia

	MVB12B							
1D_DN	*MVB1 2B*	2.8 Mb	Loss	9q33.3q34.11 128.460-131.260 (*de novo*)	1 yr	ID	Brachycephaly, microcephaly	Feeding difficulties at infancy
2D_DN	*MVB1 2B*	4.1 Mb	Loss	9q33.3q34.11 128.870 to 132.995 Mb (*de novo*)	1 yr	N/A	N/A	
3G	*MVB1 2B*	683 Kb	Gain	129.218 to 129.902 Mb	N/A	Developmental delay and/or ID	N/A	N/A
3D_DN	*MVB1 2B*	1.2 Mb	Loss	9q33.3 128.652 to 129.871 Mb (*de novo*)	< 1 yr	N/A	N/A	
4D_DN	*MVB1 2B*	4.1 Mb	Loss	9q33.3q34.11 127.213 to 131.263 Mb (*de novo*)	9 yrs	N/A	N/A	
4G_DN	*MVB1 2B*	471 Kb, 703 Kb	Gains	9q33.3 128.772 to 129.243 Mb 9q34.11 130.898 to 131.601 Mb (both *de novo*)	N/A	ID		Unaffected niece has 9q33.3 gain.
5D_DN	*MVB1 2B*	330 Kb	Loss	9q33.3 129.156 to 129.490 Mb (*de novo*)	N/A	Global Developmental Delay	-	
5G_DN	*MVB1 2B*	839 Kb	Gain	9q33.3 128.653 to 129.491 Mb (*de novo*)	15 yrs	Learning disability		Tourette syndrome, ADHD
6D_DN	*MVB1 2B*	470 Kb, 700 Kb	Loss	9q33.3 128.772 to 129.243 Mb 9q34.11 130.898 to 131.601 (Both *de novo*)	N/A	Developmental Delay, ID	-	Autism
6G_DN	*MVB1 2B*	1.9 Mb 7.5 Mb	Gains	9q33q34.11, 128.432 to 130.352 Mb 9q34.11q34.3	2 yrs	N/A	N/A	

				130.825 to 138.309 Mb (both *de novo*)				
7D_DN	*MVB12B*	980 Kb	Loss	9q33.3 129.136 to 130.120 Mb (de novo)	5 yrs	Developmental delay	-	Patellar aplasia
12D_DN	*MVB12B*	3.6 Mb	Loss	9q33.3q34.11 127.818-131.400 Mb (*de novo*)	18 yrs	Developmental delay	-	Seizures; deletion includes *STXBP1*
	PP1R9A							
1D	*PPP1R9A*	8.8 Mb	Loss	93.184 to 102.043 Mb	N/A	Developmental delay and/or ID	N/A	N/A
3D_DN	*PPP1R9A*	6.8 Mb	Loss	7q21.3q22.1 94.174 to 10.101 Mb (*de novo*)	< 1 yr	ID, speech delay	Epicanthus, posteriorly rotated ears	Ectrodactyly
4D	*PPP1R9A*	5.9 Mb	Loss	7q21.2q21.3 91.997 to 97.905 Mb	N/A	ID	Micrognathia	Short stature, congenital hip dislocation, short stature, sensorineural hearing loss
5D	*PPP1R9A*	5.8 Mb	Loss	93.891 to 99.735 Mb	N/A	Developmental delay and/or ID	N/A	N/A
6D	*PPP1R9A*	5.8 Mb	Loss	89.836 to 95.635 Mb	N/A	Developmental delay and/or ID	N/A	N/A
7D	*PPP1R9A*	2 Mb	Loss	7q21.3 92.943 to 94.931 Mb	12 yrs	-	Triangular facies, broad forehead, thin lips.	Myoclonus dystonia, short stature, failure to thrive, anxiety, obsessive-compulsive behavior.
9D	*PPP1*	1.3	Loss	7q21.3	2 yrs	-		Short

	R9A	Mb		94.909 to 96.189 Mb				stature, sensorineural hearing loss, congenital hip dislocation
10D	*PPP1 R9A*	1.1 Mb	Loss	93.926 to 95.027 Mb	N/A	Developmental delay and/or ID	N/A	N/A
11D_DN	*PPP1 R9A*	201 Kb	Loss	7q21.3 94.823 to 95.024 Mb (*de novo*)	3 yrs	Speech delay	-	Repetitive behaviors and sensory sensitivities consistent with autism spectrum disorder
12D	*PPP1 R9A*	2.1 Mb	Loss	7q21.3 92.992 Mb to 95.058 Mb (Paternal)	21 months	-	Ear pit, helix	Hyperplasia of right leg; Father has tremors due to *SGCE* deletion.
13D_DN	*PPP1 R9A*	4.8 Mb	Loss	7q21.2q21.3 92.388 to 97.197 Mb (*de novo*)	4 yrs	Fine and gross motor delay, speech delay, ID	Microcephaly	Short stature, ADHD, hypotonia, autism

Table S8. Primer sequences used in this study for relative expression (to ACTB or MED13) quantification using quantitative rt-PCR of critical exons located within *MVB12B*, *PPP1R9A*, and *GIT1* gene.

Primer Name	Sequence (5'-3')
MVB12B-F	TTC ATC CCA ATT CAG GAG AC
MVB12B-R	CAT GAT CCG AAT GTC ACA AA
PPP1R9A-F	AGC AGG TTT CTC ACT GGT TA
PPP1R9A-R	GAT GCT GTC ATT CCA AGA GC
GIT1-F	GCC TTG ACT TAT CCG AAT TG
GIT1-R	ACC TCG TCA TAC ACG TCC A
ACTB-F	ATT GCC GAC AGG ATG CAG A
ACTB-R	GAG TAC TTG CGC TCA GGA GGA
MED13-F	CCG CAT CCT GAT GTG TCT GA
MED13-R	TTG CAG GTG GAT ACG TGA CT

References:

1. Kearney, H.M. *et al.* American College of Medical Genetics standards and guidelines for interpretation and reporting of postnatal constitutional copy number variants. *Genet Med* **13**, 680-5 (2011).
2. Bierut, L.J. *et al.* A genome-wide association study of alcohol dependence. *Proc Natl Acad Sci U S A* **107**, 5082-7 (2010).
3. Coviello, A.D. *et al.* A genome-wide association meta-analysis of circulating sex hormone-binding globulin reveals multiple Loci implicated in sex steroid hormone regulation. *PLoS Genet* **8**, e1002805 (2012).
4. Bierut, L.J. *et al.* Novel genes identified in a high-density genome wide association study for nicotine dependence. *Hum Mol Genet* **16**, 24-35 (2007).
5. Verhoeven, V.J. *et al.* Genome-wide meta-analyses of multiancestry cohorts identify multiple new susceptibility loci for refractive error and myopia. *Nat Genet* **45**, 314-8 (2013).
6. Stewart, A.F. *et al.* Kinesin family member 6 variant Trp719Arg does not associate with angiographically defined coronary artery disease in the Ottawa Heart Genomics Study. *J Am Coll Cardiol* **53**, 1471-2 (2009).
7. Krawczak, M. *et al.* PopGen: population-based recruitment of patients and controls for the analysis of complex genotype-phenotype relationships. *Community Genet* **9**, 55-61 (2006).
8. Coe, B.P. *et al.* Refining analyses of copy number variation identifies specific genes associated with developmental delay. *Nat Genet* **46**, 1063-71 (2014).
9. Uddin, M. *et al.* Brain-expressed exons under purifying selection are enriched for de novo mutations in autism spectrum disorder. *Nat Genet* **46**, 742-7 (2014).
10. Genomes Project, C. *et al.* An integrated map of genetic variation from 1,092 human genomes. *Nature* **491**, 56-65 (2012).
11. Kim, M.S. *et al.* A draft map of the human proteome. *Nature* **509**, 575-81 (2014).
12. Langfelder, P. & Horvath, S. WGCNA: an R package for weighted correlation network analysis. *BMC Bioinformatics* **9**, 559 (2008).
13. Langfelder, P. & Horvath, S. Fast R Functions for Robust Correlations and Hierarchical Clustering. *J Stat Softw* **46**(2012).
14. Zhang, B. & Horvath, S. A general framework for weighted gene co-expression network analysis. *Stat Appl Genet Mol Biol* **4**, Article17 (2005).
